# Optimization of extracellular vesicles preparation from saliva of head and neck cancer patients

**DOI:** 10.1038/s41598-023-50610-6

**Published:** 2024-01-10

**Authors:** Luisa Tengler, Moritz Tiedtke, Julia Schütz, Karen Bieback, Stefanie Uhlig, Marie-Nicole Theodoraki, Katja Nitschke, Thomas Stefan Worst, Elena Seiz, Claudia Scherl, Nicole Rotter, Sonja Ludwig

**Affiliations:** 1 Department of Otorhinolaryngology, Head and Neck Surgery, University Hospital Mannheim, Medical Faculty Mannheim, Heidelberg University, Mannheim, Germany; 2 Institute of Transfusion Medicine and Immunology, Medical Faculty Mannheim, Heidelberg University, German Red Cross Blood Service, Baden‑Württemberg‑Hessen, Mannheim, Germany; 3 Department of Otorhinolaryngology, Head and Neck Surgery, Klinikum Rechts Der Isar, Technical University Munich, Munich, Germany; 4 Department of Urology and Urosurgery, Medical Faculty Mannheim, University Hospital Mannheim, University of Heidelberg, Mannheim, Germany

**Keywords:** Head and neck cancer, Biomarkers

## Abstract

Small extracellular vesicles from saliva (SEVs) have high potential as biomarkers in Head and Neck cancer (HNC). However, there is no common consensus on the ideal method for their isolation. This study compared different ultracentrifugation (UC) methods (durations and + /− additional purification) with size exclusion chromatography (SEC) and investigated the potential of SEVs as diagnostic biomarkers and their biological activity on NK and CD8^+^ T cells. SEVs from 19 HNC patients and 8 healthy donors (HDs) were thoroughly characterized. Transmission electron microscopy confirmed the isolation of vesicles by all methods. The average size determined via nanoparticle-tracking analysis was smaller for SEVs isolated by SEC than UC. The highest particle-to-protein yield was achieved by UC (3 h + 3 h) (UC_opt_) and SEC. However, SEC yielded considerably fewer SEVs. Comparing the surface marker cargo, SEVs isolated by UC_opt_ from HNC patients carried more PD-L1, FasL, and TGF-β than SEVs from HDs. These levels correlated with tumor stage and HPV status. SEVs downregulated NKG2D expression on primary NK cells. HNC SEVs accelerated CD8^+^ T cell death compared to HD SEVs. This study suggests that UC_opt_ is preferable when isolation of a high particle-to-protein load is required. Especially PD-L1 and FasL on SEVs hold substantial potential as diagnostic biomarkers.

## Introduction

Head and neck cancers (HNCs) account for one of the most common tumor entities worldwide with around 880,000 new cases per year. Most HNCs are locally progressed at the time of diagnosis, leading to frequent therapy failures and recurrences^1^. Thus, there is an urgent need for novel minimally invasive biomarkers aiding early tumour detection. Small extracellular vesicles (EVs) from biofluids, including saliva and plasma, are promising candidates for liquid biopsy applications to detect early tumors, residual disease, or recurrences.

Small EVs from the plasma of HNC patients have been shown to reflect the disease activity and tumor stage of HNCs, underlining their biomarker potential in HNCs^2^. Small EVs are nano-scaled, lipid bi-layered particles ranging from 30 to 150 nm in size and mirror the cargo of their parent cells^3^. As a source of small EVs, biofluids contain a mixture of tumor-derived EVs (TEVs) and EVs from other sources, i.e. hematopoietic cells and other body cells (non-TEVs). Due to the particular exposure of saliva to the head and neck region, salivary small EVs (SEVs) have an exceptionally high proportion of TEVs from local HNCs (i.e. oral cavity, oropharynx) compared to small EVs from plasma^4^.

In addition to being enriched with TEVs, saliva as a diagnostic biofluid has several advantages over blood plasma or serum: saliva collection is non-invasive, cost-effective, and requires little training, allowing repeated sampling, even of complex study populations (e.g., anxious patients, children)^5^. However, the complex mixture of saliva has impeded the development of reliable isolation techniques for SEVs. Salivary composition is highly dependent on salivary gland discharge, microorganisms, tissue, immune cells, and cell debris^6^. Undesirable by-products include highly-abundant proline-rich peptides, α-amylase and mucins^5^, and can potentially mask low-abundant, relevant proteins in the saliva ^7,8^.

So far, several techniques for preparing EVs from saliva have been described: The most common are ultracentrifugation^9^, size exclusion chromatography (SEC)^10^, density gradient centrifugation^11^, and precipitation (i.e. ExoQuick-TC™^12^).

In this study, we aim to optimize the preparation of SEVs from healthy donors (HDs) and HNC patients with high purity and vesicle integrity and then compare the two standard techniques, ultracentrifugation and SEC, for diagnostic and functional applications in HNC.

Previous studies have established that the cargo of small EVs from HNC plasma (particularly in advanced stages) suppresses anti-tumor immunity, contributing to immune escape^2,13–15^. Whereas several studies have previously examined the diagnostic and functional potential of small EVs from the plasma of HNC patients, limited research studies have investigated SEVs and mainly focused on the value of RNAs in SEVs as liquid biomarkers for HNC^4,16–18^ or other cancer subtypes^18^. To account for this, we assessed the abundance of immunomodulatory proteins and performed functional experiments comparing SEVs from different isolation techniques regarding the regulation of NKG2D receptor expression on primary NK cells and apoptosis in primary CD8^+^ T cells.

## Material and methods

### Sample collection

Saliva specimens were collected from HNC patients (N = 19) as well as age- and gender-matched healthy donors (N = 8) at the Department of Otorhinolaryngology, Head and Neck Surgery of the University Hospital Mannheim from 2021 until 2023 (Suppl. Tables 1 and 2). Informed consent was obtained from participants before inclusion in the study. The local ethics committee of the Medical Faculty Mannheim, Heidelberg University (Ethics Committee II) approved the study (2021–552) in accordance with good clinical practice guidelines and the Declaration of Helsinki.

Saliva collection was only pursued in fasting patients within a standard time frame (7–10 am) and using 5–6 salivettes (Sarstedt). The patients were instructed to chew on each cotton swab for 1 min until thoroughly soaked and the salivette was immediately kept on ice. Briefly, the salivettes were centrifuged at 1000 g for 2 min, to retrieve the saliva. The saliva was pooled and stored in 1 mL aliquots at −80 °C.

### EV isolation via size-exclusion chromatography and ultracentrifugation

Two different methods for EV isolation were employed following the scheme in Suppl. Figure 1. To isolate small EVs from patient saliva, size-exclusion chromatography (SEC) was performed. Briefly, samples were thawed and differentially centrifuged at 3000 g for 10 min at RT and 14,000 g for 30 min at 4 °C. Subsequently, the samples were ultrafiltered through a 0.22 µm filter (Millipore) to deplete residual cellular fragments. The SEC columns were self-made using Sepharose CL-2B (Cytiva) as described previously^19^. 1 mL of pre-cleared saliva was applied onto the column bed and subsequently eluted with 1 mL PBS and fraction #4 with the highest yield of SEVs was collected.

Alternatively, after differential centrifugation, samples were diluted 1:1 with PBS and filtered through a 0.22 µm filter (Millipore). The samples were filled into thick-walled polycarbonate tubes (Beckman Coulter) and centrifuged at 100,000 g for 3 h, 6 h or 12 h at 4 °C in an Optima XPN-100 ultracentrifuge using the Type 70 Ti rotor (k-factor: 69). Additionally, a purification step (PBS) was included to obtain a higher purity (3h + 3h = UC_opt_, 6h + 3h, 12h + 3h). The supernatant after the first centrifugation was collected (SUP), the EV pellet was resuspended with another 2 mL of PBS, and the centrifugation step was repeated.

### Characterization of the SEVs

TEM of SEVs from HDs and HNC patients was performed at the Electron Microscopy Core Facility of Heidelberg University as previously described ^20^.

SEVs were diluted up to 1:3000 in PBS and measured at room temperature (RT) using the Nanoparticle tracking analyzer (NTA) ZetaView® TWIN (Particle Metrix). Particle size and concentration were determined using the ZetaView Software (Particle Metrix Version 8.05.11 SP1 and SP2, Sensitivity 85%, Shutter 100, Min Area 15, Max Area 2000, Min Brightness 20, Trace Length 30, 11 positions, 2 cycles).

For protein quantification, Pierce™ BCA protein assay (Thermo Scientific) was performed according to the manufacturer’s instructions.

### SDS-PAGE and western blots

SEV samples (5–10 μg) in non-reducing (CD63 and CD81) or reducing sample buffer were separated on 4–20% polyacrylamide gels (Bio-Rad) and transferred onto nitrocellulose membranes (Bio-Rad). Briefly, after blocking, the membrane was incubated with the following primary antibodies overnight at 4 °C according to manufacturer’s instructions: anti-CD63 (#10628D, 1:250), anti-CD9 (#10626D, 1:500), anti-CD81 (#10630D, 1:500), anti-TSG101 (#PA5-31,260, 1:500) from Invitrogen; anti-PD-L1 (#13,684, 1:1000), anti-amylase (#3796,1:1000), anti-TGF-β (#3711, 1:1000) from Cell Signaling Technology; anti-CTLA-4 (#TA810204, 1:500) from OriGene; anti-TRAIL (#ab2056, 1:500) from Abcam. After washing, HRP-conjugated secondary antibodies (IgG Rabbit anti-Mouse, 1:10,000 or IgG Goat anti-Rabbit, 1:10,000; Thermo Scientific) were incubated for 1 h at RT. The chemiluminescence signal was elicited by SuperSignal™ West Dura™ Chemiluminescence Substrate. Images were acquired with the iBright Imager. To compare the total protein content of SEVs, 35 µL of the samples were loaded and gels were stained using Coomassie blue (Thermo Scientific).

### Flow cytometry of SEV surface markers

To analyze the content of immunomodulatory proteins on the surface of SEVs, a spectral flow cytometer with an enhanced small particle detection option (Cytek® Northern Lights™) at the FlowCore Mannheim was used. SEVs were diluted (1:20 in 100 µL PBS) and stained with 1 µL fluorescently-labeled antibodies for tetraspanins (CD9-, CD63-, CD81-FITC; BioLegend) and immunomodulatory proteins (PD-L1-PE, CTLA-4-APC, FasL-PE, TGF-β-APC, Tim3-BV421, LAG-3-BV421; BioLegend) for 30 min at 4 °C. Afterwards EVs were diluted 1:4 in PBS and 50 µL of sample was acquired. As negative controls, PBS only and PBS with antibodies were used. To prove that the signal was related to SEVs, we also disrupted the lipid bilayer of the vesicles using 0.5% sodium dodecyl sulfate (SDS). Next, EVs were gated according to ApogeeMix beads (#1527) to exclude particles or aggregates that are larger than 500 nm polystyrene beads (Suppl. Figure 2). Additionally, isotype and SDS controls were examined to exclude unspecific binding of the antibodies. The count of positive events for immunomodulatory proteins in the EV marker-positive population was calculated using FlowJo and normalized to the volume of 50 µL.

### PBMC and T cell isolation

PBMCs were isolated from buffy coats of healthy donors (from local German Red Cross blood donation service) via density gradient centrifugation using Ficoll-Paque™. To isolate CD8^+^ lymphocytes, T Cell Isolation Kits (Miltenyi) were used according to manufacturers’ instructions. Purity was confirmed by staining with anti-CD8-PE (1:100, BioLegend) antibodies for 30 min at 4 °C and measuring with the BD FACS Canto II.

### T cell apoptosis analysis

To assess T cell apoptosis, Annexin V assays (eBioscience) were performed. CD8^+^ primary T cells were seeded at a density of 10^6^ cells/mL in 96-well plates (100,000 cells/well) in AIM-V medium. Primary T cells were activated with CD3/CD28 antibodies (25 μL/mL, Stemcell) and IL-2 (150 U/mL, Peprotech) for 24 h. The T cells were treated with 50 µL PBS as vehicle control, 50 µL of SEC-isolated SEVs, 50 µL of UC_opt_-isolated SEVs (resuspension to the input volume, to retrieve the SEV amount of 50 µL saliva), and 50 µL of supernatant from UC (SUP) for another 24 h. Boiled, dead T cells were used as an Annexin V-binding control. All cells were stained with Annexin V-FITC/-APC (1:100) for 15 min at RT, followed by washing and staining with propidium iodide (1:200). At least 30,000 cells per sample were analyzed with a BD FACS Canto II.

As an alternative method, caspase activity was measured using CaspaseGlo 3/7 Assays (Promega) on SEV-treated T cells. CD8^+^ primary T cells (10,000 cells/well) were seeded in 96-well plates in 50 µL AIM-V and were treated with 50 µL SEVs or PBS for 24 h. Then, 100 µL assay reagent was added to the cells, incubated for 1 h at RT, and luminescence was read out with the Tecan plate reader.

### NKG2D assay

To analyze the effect of SEVs on the activity of NK cells, the downregulation of the NKG2D receptor on NK cells was analyzed. Freshly isolated PBMCs were seeded in AIM-V medium (500,000 cells/well) in 96-well plates and treated with 50 µL PBS as vehicle control, 50 µL of SEC-isolated SEVs, 50 µL of UC_opt_-isolated SEVs, and 50 µL of supernatant from UC (SUP). After 24 h, PBMCs were incubated with F_c_R-blocking reagent (1:100) for 10 min at 4 °C and stained using CD3-FITC (1:50), CD56-PE (1:50) and NKG2D-APC (1:50) antibodies from BioLegend to measure the expression of the receptor on NK cells (CD3^-^CD56^+^).

### Statistics

Results were graphed and analyzed using GraphPad Prism (9.4.1). As nonparametric tests, Kruskal–Wallis or Mann–Whitney tests were used. If a significant result was obtained with Kruskal–Wallis tests, post hoc tests were conducted for pairwise comparisons. In the case of small sample sizes and many groups, *p*-values were not adjusted for multiple testing (Dunn's multiple comparisons test). For the comparison of the functional assays, Friedmann tests for matched groups were employed. Quantitative data is presented as mean ± SD (standard deviation) unless otherwise specified. In general, a p-value less than 0.05 was considered statistically significant.

## Results

### Comparison of ultracentrifugation and size exclusion chromatography for preparation of EVs from saliva

First, we analyzed which preparation technique of SEVs is the optimal condition for diagnostic and functional applications with a focus on achieving high recovery and maintaining vesicle integrity and functionality.

Saliva was collected from HDs and HNC patients using cotton rolls. Following differential centrifugation and ultrafiltration, SEVs were either isolated by UC with and without the purification step or SEC according to the scheme in Suppl. Figure 1. As plasma and saliva are distinct biofluids and the SEC method was optimized for plasma application^19^, we also verified the ideal SEC fraction for saliva with the highest content of EVs (Suppl. Figure 3). In line with the plasma samples, most vesicles were eluted in fraction 4 and the majority of proteins eluted in fraction 6.

**Figure 1:**
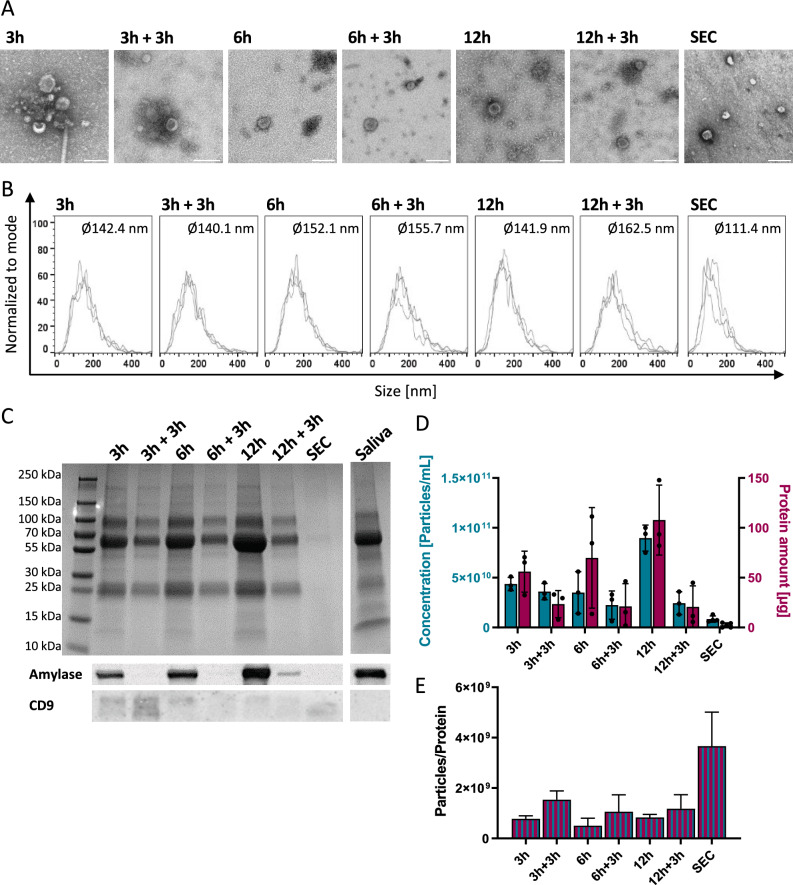
Characterization of the SEVs from healthy donors (HDs). For the preparation of SEVs, ultracentrifugation (UC) was performed using various centrifugation durations without (3h, 6h, 12h) or with an additional purification step (3h + 3h, 6h + 3h, 12h + 3h). UC was compared to the Sepharose-based size exclusion chromatography (SEC) approach. (**A**) Transmission electron microscope (TEM) pictures visualize the isolated vesicles. The scale bar represents 100 nm. (**B**) Size distribution histograms of the isolated particles detected by Nanoparticle tracking analysis (NTA) (Ø represents the mean of the median diameter of 3 HDs). (**C**) Coomassie blue staining visualizes the total protein load of the SEVs and whole saliva (total volume of 35 µL loaded per lane) and the Western blots confirm the presence of α-amylase and CD9 (5µg per lane). Original Western blot images and the Coomassie-stained gel are available in Suppl. Figure 4 (**D**) Direct comparison of particle concentration (particles/mL) and total protein amount (μg), and (**E**) particle-to-protein ratio for the different preparation methods. The graphs show column bars displaying the means and SD of three healthy individuals.

SEVs obtained from UC and SEC showed the typical vesicular morphology and size range in TEM (Fig. 1A). Samples isolated via UC with the purification step or SEC appeared cleaner in TEM with fewer protein aggregates compared to samples isolated via UC without purification. The analysis of particle sizes using NTA indicated that SEVs isolated with UC (Ø 141.9–162.5 nm) are larger than SEC-isolated SEVs (Ø 111.4 nm) (Fig. 1B). Larger average sizes were observed with longer UC (≥ 6h). Although SEC appears to be a cleaner preparation technique for smaller sizes, it also drastically reduces the total number of particles (67% lower yield). Coomassie blue staining of SDS-PAGE-separated samples revealed a high protein load for whole saliva and UC samples without purification step, particularly for sizes between 55 and 70 kDa (Fig. 1C). Western blots showed that the purification decreased the amount of amylase while increasing the content of the EV-marker CD9. Interestingly, in SEC samples, amylase was not detectable, while a lower CD9 amount (compared to UC) was observed (Fig. 1C, Suppl. Figure 4). Comparing particle and protein yield, a longer duration of ultracentrifugation (12h vs. 3h) increased the particle numbers and the total protein amount. Purification steps and SEC reduced the total particle and protein amount, showing the lowest particle numbers for SEC (Fig. 1D). The introduction of a purification step increased the EV purity in the UC samples by reducing the protein content and increasing the particle-to-protein ratio (Fig. 1E). Therefore, in the second part of the study addressing the diagnostic utility and functional effects of SEVs from HNC patients, we used UC with the purification step (3h + 3h) (UC_opt_).

### Characterization of SEVs from HNC patients and HDs

**Figure 2:**
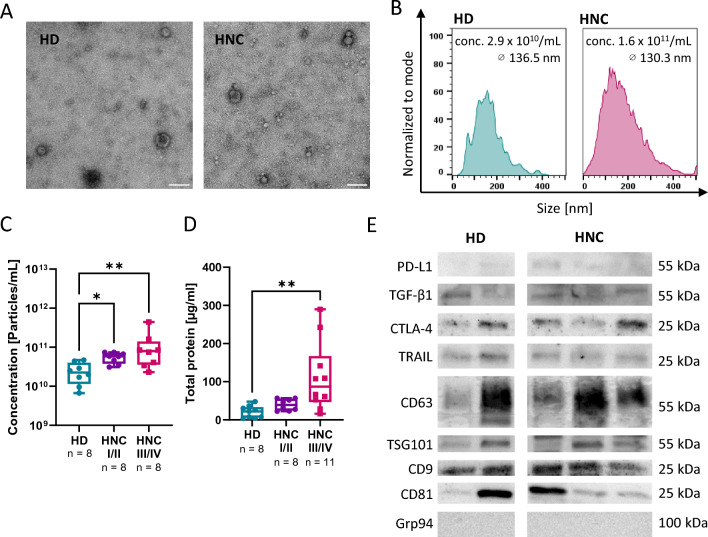
Morphological and technical features of SEVs from HNC patients and HDs. (**A**) Representative TEM images of the SEVs and (**B**) NTA-detected size distribution histograms of one representative HNC patient and HD. (**C**) Particle concentration and (**D**) total protein content of the SEVs from HDs and HNC patients with early (I/II) and advanced (III/IV) stage tumors. (**E**) Protein content of EV-defining markers (tetraspanins, TSG101) and immunoregulatory markers (PD-L1, TGF-β1, CTLA-4, TRAIL). Original Western blot images are available in Suppl. Figure 5. A p-value below 0.05 was considered significant (*p < 0.05, **p < *0.01*). n: number of patients/controls.

TEM examination showed that there were no differences in the structural morphology and diameter of SEVs between HNCs and HDs (Fig. 2A/B). However, significantly higher particle concentrations and total SEV protein load were observed in HNC patients than in HDs (*p* < *0.05*; Fig. 2C/D), which even increased in advanced HNCs.

### PD-L1, FasL, and TGF-β as potential diagnostic markers on SEVs for HNC

Western blots revealed that SEV samples from HNC patients contain higher levels of the immunoregulatory markers PD-L1 and CTLA-4 than HDs, while typical small EV-markers, TSG101, and tetraspanins, were present for both (Fig. 2E, Suppl. Figure 5).

**Figure 3:**
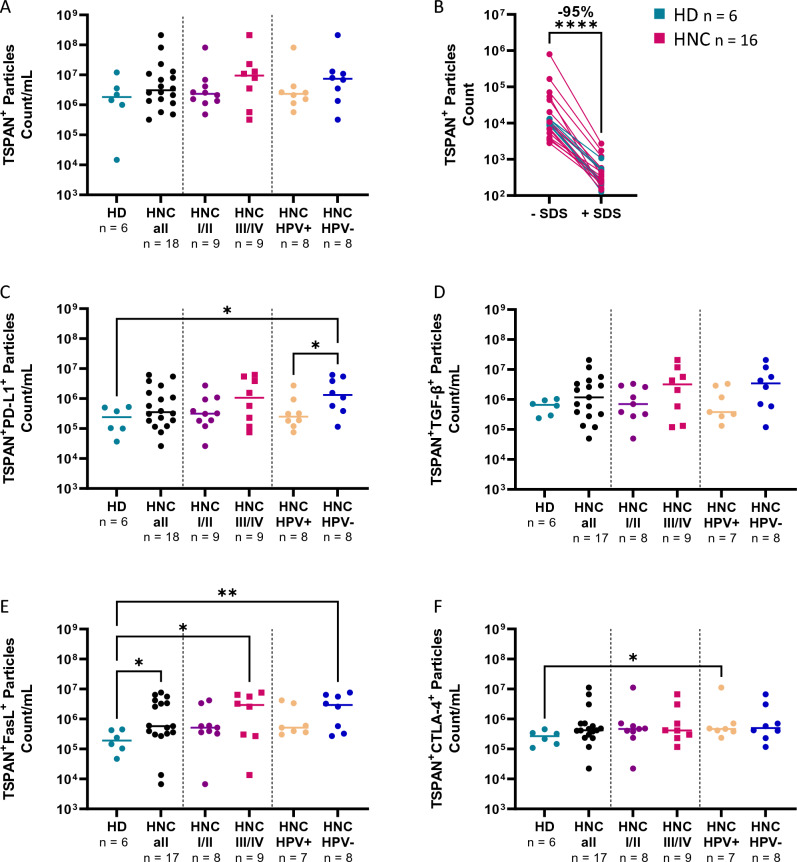
Surface markers on SEVs from HNC patients and HDs. (**A**) Count of particles stained for tetraspanins (TSPAN; CD9, CD63, CD81). (**B**) Upon exposition to SDS, the TSPAN signal decreased significantly. The co-localization of tetraspanin and PD-L1 (**C**), TGF-β (**D**), FasL (**E**), and CTLA-4 (**F**) on the surface of SEVs. The immune modulatory proteins, PD-L1 and FasL, are elevated in advanced and HPV-negative tumor stages. The bar represents the median (*p < 0.05, **p < 0.01, ****p < *0.0001*). n: number of patients/controls.

Full spectrum flow cytometry confirmed that SEV samples from late-stage HNC patients contained more tetraspanin-positive (CD63, CD81, and CD9) particles than HDs (Fig. 3A). Upon exposure to SDS, the tetraspanin-positive signal decreased (−95%), confirming the analysis of vesicular structures (Fig. 3B).

The immunoregulatory markers, PD-L1, FasL, and TGF-β, showed higher levels in HNC patients than in HDs. Interestingly, levels of PD-L1, FasL, and TGF-β were more pronounced in HNC patients with advanced than early stages (> 4x) and HPV-negative than HPV-positive tumors (> 3x) (Fig. 3C-F). Additionally, the median count of TSPAN^+^CTLA-4^+^ particles doubled on average for HNC samples compared to HDs, but in contrast to the other markers did not show a difference between HPV-positive and -negative tumors (Fig. 3F). However, there is considerable variability among the samples.

### Functional properties of SEVs on immune cells

**Figure 4:**
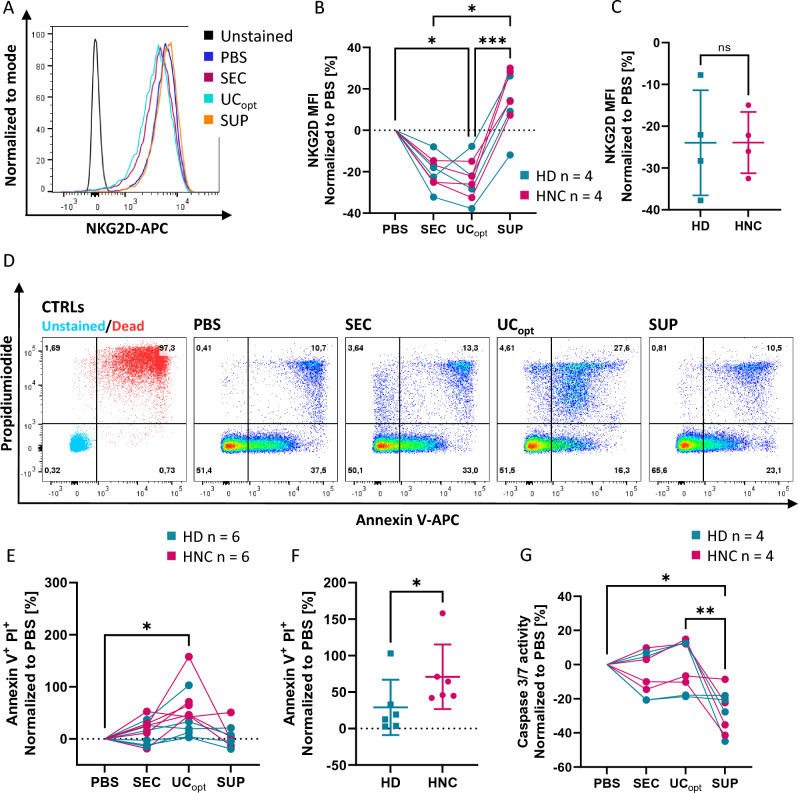
Functional comparison between SEC and UC_opt_. (**A**–**C**) PBMCs and (**D**–**G**) primary CD8^+^ T cells were co-incubated with PBS (no SEVs), SEC (SEVs isolated via SEC), UC_opt,_ and SUP (SEVs isolated by UC or the supernatant). (**A**) Representative graph for NKG2D staining on NK cells (gated from PBMCs) treated with one HNC sample. The histograms are normalized to the mode. (**B**) Line graph comparing the impact of different treatments on NKG2D expression normalized to the PBS control. (**C**) Dot plot comparing the downregulation of NKG2D by UC_opt_-derived samples from HNC and HD. (**D**) Gating strategy for Annexin V and PI staining. (**E**) Line graph comparing the effect of different treatments on the frequency of Annexin V^+^PI^+^ T cells normalized to the PBS control. (**F**) Dot plot demonstrating significant (*p* < *0.05*) differences between the frequency of Annexin V^+^ and PI^+^ cells resulting from treatment with UC-derived samples from HNC patients and HDs. (**G**) Line graph comparing the impact of different treatments on the caspase 3/7 activity, normalized to the PBS control. A *p*-value below 0.05 was considered significant (*p < 0.05, **p < 0.01, ***p < *0.001).* n: number of patients/controls.

The functional assays demonstrated that SEVs significantly downregulated the expression of the NKG2D receptor on NK cells (Fig. 4A,B). SEVs isolated via UC_opt_ more effectively reduced the NKG2D receptor compared to SEVs isolated via SEC. In contrast, the supernatant collected after UC_opt_ somewhat increased NKG2D expression. However, there was no difference between HD and HNC SEVs on NKG2D expression (Fig. 4C).

Since TRAIL, FasL, and PD-L1 were frequently detected in SEVs from patients, the induction of apoptosis in CD8^+^ primary T cells was assessed. Surprisingly, upon coincubation with SEVs isolated from patients via UC, the frequency of Annexin V^+^ apoptotic cells did not increase (Fig. 4D). Instead, SEVs from patients significantly increased double-positive Annexin V^+^ and PI^+^ dead cells in comparison to PBS and HDs (*p* < *0.05*) (Fig. 4E-F). Caspase 3/7 activity was not elevated in CD8^+^ primary T cells exposed to SEVs (Fig. 4G). The supernatant collected after UC decreased the CD8^+^ T cell apoptosis.

## Discussion

While saliva could potentially be a suitable liquid biomarker within screening programs due to its good accessibility and low invasiveness, the use of saliva and its SEVs is still an underutilized diagnostic method. Salivary composition is highly dependent on circadian rhythms and medications ^21^, which requires a well-standardized sampling method and, in contrast to blood sampling, a higher level of patient cooperation.

In the past, the preparation of SEVs has been considered challenging due to the complexity of saliva and its high viscosity. The overall goal of EV-isolation techniques is to enable the most efficient and purest preparation of intact and functional EVs ^22^. While SEC seems to yield cleaner preparations for smaller sizes, it also significantly reduces the total particle count. Assuming that the majority of the protein components are high-abundant proteins in the saliva, the highest particle preparation efficacy (high particle load, minimal contamination) was achieved in UC with the purification step (3h + 3h) (UC_opt_). UC-isolated vesicles are larger on average than those isolated by SEC. Compared to the mean diameter of small EVs from plasma, SEVs tend to be larger, which has been reported previously^4,12,23^.

Our findings suggest that saliva can serve as a liquid biomarker with a high diagnostic value: Factors such as particle concentration, tetraspanin surface cargo, and total protein of SEVs are elevated in patients compared to HDs, and even increase with disease progression. Interestingly, similar particle concentrations and total protein load have been observed in small EVs from plasma^2,20^. Moreover, our results indicate that the surface marker cargo of immunomodulatory proteins correlates with the tumor stage and HPV status. PD-L1, FasL, and TGF-ß may be attractive biomarker candidates but remain to be studied in larger patient cohorts.

Ultimately, in addition to assessing physical criteria and EV cargo, it is crucial to include testing for biological activity as a vital indicator of the method's efficacy in isolating intact small EVs^4,24^. According to our results, the most efficient method for SEV preparation from HDs was UC_opt_ providing a high particle-to-protein ratio with low contaminants. Previous studies on small EV isolation from blood serum or plasma made similar observations that several UC steps with washing steps are required to improve the purity of the EV samples^25^. A known limitation of UC is that high-speed ultracentrifugation destroys the integrity of EVs, which might limit their biological activity^26^. However, the functional results of our study implicate that SEVs isolated by both UC_opt_ and SEC (Fig. 4) show biological activity: SEVs from patients and HDs impair NK cell and lymphocyte functions compared to the negative control (no SEVs). SEVs isolated via UC_opt_ induce higher levels of Annexin V^+^ and PI^+^ CD8^+^ T cells than SEVs isolated by SEC (Fig. 4E/F). This difference can be attributed to the higher protein and particle concentration in UC isolated SEVs suggesting that UC leads to a higher concentration of functionally active SEVs.

As shown previously, SEVs have a primarily cancerous origin as evidenced by their CD44v3 cargo (TEV), in contrast to small EVs from plasma, which are mainly of hematopoietic origin (non-TEVs)^4^. From plasma and cell culture studies, it is well-known that TEVs are highly immunosuppressive^2,24,27,28^. The different functionality of SEVs might reflect the different compositions of EVs from saliva compared to plasma. Interestingly, the supernatant collected after UC showed a contrasting effect on immune cells compared to the isolated SEVs. This suggests that the observed impact on the immune cell functions is indeed mediated by SEVs and not soluble factors. Further studies involving larger patient cohorts, different subsets of immune cells, and different doses of SEVs are required to classify the immunological capacity of SEVs.

In summary, our work supports UC_opt_ as the preferred method for SEV isolation. Particularly, surface proteins on SEVs, like PD-L1, FasL, and TGF-β could serve as potential diagnostic tools. SEVs from both HDs and HNC patients mediate immunosuppressive functions on NK cells and T cells, which are partly not disease-specific. Further studies are needed to confirm the biomarker potential of SEVs in HNC and to gain deeper insights into the functionality of SEVs.

### Supplementary Information

Supplementary Information.

## Data Availability

The datasets generated and/or analyzed during the current study are available from the corresponding author upon reasonable request.

## References

[1] Sung H (2021). Global cancer statistics 2020: GLOBOCAN estimates of incidence and mortality worldwide for 36 cancers in 185 countries. CA: Cancer J. Clin..

[2] Ludwig S (2017). Suppression of lymphocyte functions by plasma exosomes correlates with disease activity in patients with head and neck cancer. Clin. Cancer Res. Off. J. Am. Assoc. Cancer Res..

[3] Théry, C., *et al.* Minimal information for studies of extracellular vesicles 2018 (MISEV2018): a position statement of the International Society for Extracellular Vesicles and update of the MISEV2014 guidelines. *J. Extracell. Vesicles*. **7**(1) (2018). 10.1080/20013078.2018.1535750 PMC6322352 30637094

[4] Hofmann L (2022). Cargo and functional profile of saliva-derived exosomes reveal biomarkers specific for head and neck cancer. Front. Med..

[5] Pfaffe T (2011). Diagnostic potential of saliva: current state and future applications. Clin. Chem..

[6] Humphrey SP, Williamson RT (2001). A review of saliva: normal composition, flow, and function. J. Prosthet. Dent..

[7] Krief G (2011). Improved visualization of low abundance oral fluid proteins after triple depletion of alpha amylase, albumin and IgG. Oral Dis..

[8] Deutsch O (2008). An approach to remove alpha amylase for proteomic analysis of low abundance biomarkers in human saliva. Electrophoresis.

[9] Théry, C., *et al.* Isolation and characterization of exosomes from cell culture supernatants and biological fluids. Current protocols in cell biology. **Chapter 3**: p. Unit 3.22 (2006). 10.1002/0471143030.cb0322s30 18228490

[10] Han, P., *et al.* Detection of salivary small extracellular vesicles associated inflammatory cytokines gene methylation in gingivitis. *Int. J. Mol. Sci.* 2020/08. **21**(15). 10.3390/ijms21155273 PMC7432462 32722322

[11] Iwai, K., *et al.* Isolation of human salivary extracellular vesicles by iodixanol density gradient ultracentrifugation and their characterizations. *J. Extracell. Vesicles* **5** (2016). 10.3402/jev.v5.30829 PMC4871899 27193612

[12] Zlotogorski-Hurvitz A (2015). Human saliva-derived exosomes: comparing methods of isolation. J. Histochem. Cytochem. Off. J. Histochem. Soc..

[13] Beccard, I.J., *et al.* Immune suppressive effects of plasma-derived exosome populations in head and neck cancer. *Cancers* **12**(7) (2020). 10.3390/cancers12071997 PMC7409343 32708274

[14] Jablonska, J., *et al.* Evaluation of immunoregulatory biomarkers on plasma small extracellular vesicles for disease progression and early therapeutic response in head and neck cancer. *Cells*. **11**(5) (2022). 10.3390/cells11050902 PMC8909035 35269524

[15] Benecke, L., *et al.* Isolation and analysis of tumor‑derived extracellular vesicles from head and neck squamous cell carcinoma plasma by galectin‑based glycan recognition particles. *Int. J. Oncol.* **61**(5) (2022). 10.3892/ijo.2022.5423 PMC9507089 36129151

[16] Hofmann, L., *et al.* Comparison of plasma- and saliva-derived exosomal miRNA profiles reveals diagnostic potential in head and neck cancer. *Front. Cell Dev. Biol.* **10** (2022). 10.3389/fcell.2022.971596 PMC9441766 36072342

[17] Langevin S (2017). Comprehensive microRNA-sequencing of exosomes derived from head and neck carcinoma cells in vitro reveals common secretion profiles and potential utility as salivary biomarkers. Oncotarget.

[18] Li, K., *et al.* A signature of saliva-derived exosomal small RNAs as predicting biomarker for esophageal carcinoma: a multicenter prospective study. *Mol. Cancer* **21**(1) (2022). 10.1186/s12943-022-01499-8 PMC8764835 35042519

[19] Hong C-S (2016). Isolation of biologically active and morphologically intact exosomes from plasma of patients with cancer. J. Extracell. Vesicles.

[20] Tengler, L., *et al.* Plasma-derived small extracellular vesicles unleash the angiogenic potential in head and neck cancer patients. *Mol. Med.* **29** (2023). 10.1186/s10020-023-00659-w PMC10207688 37226100

[21] Pedersen AML (2018). Salivary secretion in health and disease. J. Oral Rehabilit..

[22] Contreras, H., *et al.* Comparative study of size exclusion chromatography for isolation of small extracellular vesicle from cell-conditioned media, plasma, urine, and saliva. *Front. Nanotechnol.* **5** (2023).

[23] Nonaka T, Wong DTW (2017). Saliva-exosomics in cancer: molecular characterization of cancer-derived exosomes in saliva. Enzymes.

[24] Buzas, E.I. & E.I. Buzas, The roles of extracellular vesicles in the immune system. *Nat. Rev. Immunol.* **23**(4) (2022). 10.1038/s41577-022-00763-8 PMC9361922 35927511

[25] An M (2018). Comparison of an optimized ultracentrifugation method versus size-exclusion chromatography for isolation of exosomes from human serum. J. Proteome. Res..

[26] Jeppesen DK (2014). Comparative analysis of discrete exosome fractions obtained by differential centrifugation. J. Extracell. Vesicles.

[27] Ortiz A (2019). An interferon-driven oxysterol-based defense against tumor-derived extracellular vesicles. Cancer Cell.

[28] Theodoraki, M.-N., *et al.* CD44v3 protein-carrying tumor-derived exosomes in HNSCC patients’ plasma as potential noninvasive biomarkers of disease activity. *Oncoimmunology* **9**(1) (2020). 10.1080/2162402X.2020.1747732 PMC7153843 32313730

