# Supplementary material for: Optimization of extracellular vesicles preparation from saliva of head and neck cancer patients
Source: Sci Rep. 2024 Jan 10;14:946. doi: 10.1038/s41598-023-50610-6 (PMC10781729; doi:10.1038/s41598-023-50610-6)
Supplement: Supplementary file 1 — Supplementary Information. [file 41598_2023_50610_MOESM1_ESM.pdf]

Supplementary Table 1. Summary of the clinicopathological characteristics of the HNC patients (N = 19).

	n	%		n	%
**Age**			**Tumor size**		
Median	65		*T1*	0	0
**Sex**			*T2*	12	63.2
Male	17	89.5	*T3*	3	15.8
Female	2	10.5	*T4*	4	21.1
**Tumor site**			**Lymph node status**		
Oral cavity	3	15.8	*N0*	7	36.8
Oropharynx	14	73.7	*N1*	6	31.6
Hypopharynx	2	10.5	*N2*	1	5.3
**HPV status⁺**			*N3*	5	26.3
Negative	9	47.4	**Metastases**		
Positive	8	42.1	*M1*	2	10.5
n/a	2	10.5	**UICC stages***		
**Smoking**			*I*	4	21.1
Never	2	10.5	*II*	5	26.3
Former	8	42.1	*III*	4	21.1
Current	9	47.4	*IV*	6	31.6
**Alcohol consumption**			**Therapy**		
Never	2	10.5	*Surgery*	5	26.3
Former	4	21.1	*Surgery plus CRT*	6	31.6
Current	12	63.2	*Surgery plus RT*	3	15.8
n/a	1	5.3	*Primary CRT*	3	15.8
			Palliative RT	2	10.5

n: number of patients, n/a: not available, CRT: chemoradiotherapy, RT: radiotherapy, ⁺p16 positivity, *International Union Against Cancer (UICC) classification 8th edition

Supplementary Table 2. Clinicopathological characteristics of the HNC patients

ID	Age	Sex	HPV status+	Tumor site	UICC stage*	Tumor size (T)	Lymph node status (N)	Metastases (M)	Smoking	Alcohol consumption	Therapy
1	72	f	-	OPSCC	III	3	0	0	+	+	Surgery + RT
2	58	m	-	OPSCC	IVB	2	3b	0	+	+	Surgery + CRT
3	47	m	+	OPSCC	I	2	1	0	+	+	Surgery + RT
4	69	m	+	OSCC	II	2	0	0	+	+	Surgery
5	64	m	-	OPSCC	II	2	0	0	ex	ex	Primary CRT
6	59	m	-	OPSCC	IVC	2	3b	1	+	ex	Palliative RT
7	71	m	-	OPSCC	IVB	2	3b	0	ex	+	Surgery
8	61	m	+	OPSCC	II	3	1	0	+	+	Primary CRT
9	56	m	+	OPSCC	II	2	2c	0	+	-	Surgery + CRT
10	65	m	-	OPSCC	III	2	1	0	-	+	Surgery + CRT
11	65	f	+	OPSCC	III	4	0	0	ex	+	Surgery + CRT
12	70	m	+	OPSCC	I	2	1	0	-	-	Surgery + CRT
13	62	m	-	OSCC	II	2	0	0	ex	ex	Surgery
14	56	m	n/a	OSCC	III	3	0	0	+	+	Surgery + RT
15	77	m	n/a	HPSCC	IVB	4a	3b	0	ex	ex	Surgery
16	68	m	-	OPSCC	IVB	4b	0	0	+	+	Primary CRT
17	71	m	-	HPSCC	IVC	4b	3b	1	ex	n/a	Palliative RT
18	70	m	+	OPSCC	I	2	1	0	ex	+	Surgery + CRT
19	61	m	+	OPSCC	I	2	1	0	ex	+	Surgery

m: male, f: female, + : positive/yes/current, - : negative/no/never, n/a: not available, OPSCC: oropharyngeal squamous cell carcinoma, OSCC: oral squamous cell carcinoma, HPSCC: hypopharyngeal squamous cell carcinoma, CRT: chemoradiotherapy, RT: radiotherapy, +p16 positivity, *International Union Against Cancer (UICC) classification 8th edition

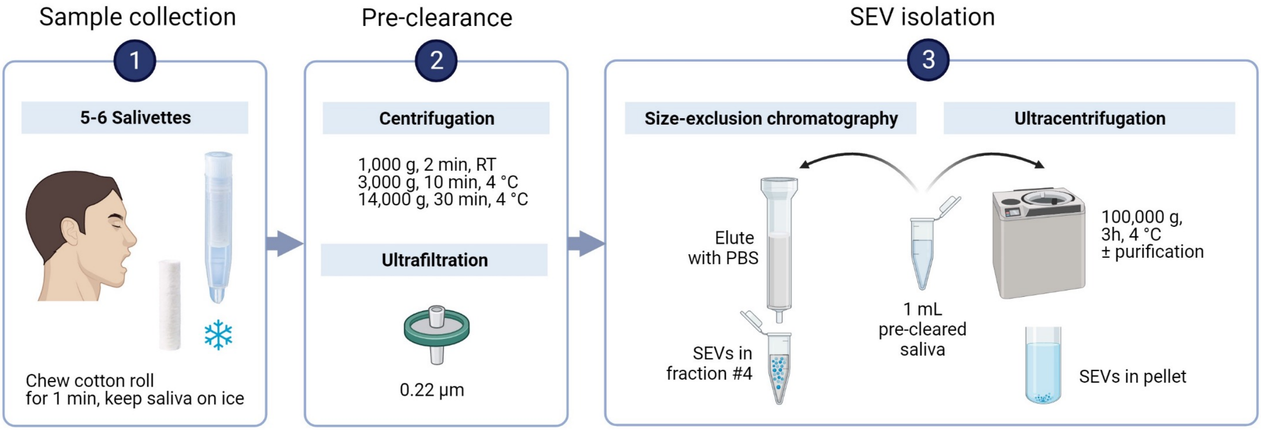

Supplementary Fig. 1. SEV isolation scheme. Saliva samples are collected using salivettes. The patients chew on each cotton swab for 1 min and the salivette is immediately kept on ice. For pre-clearance, the saliva is differentially centrifuged and ultrafiltered. SEVs are then isolated via size-exclusion chromatography or via ultracentrifugation including a purification step. Created with BioRender.com.

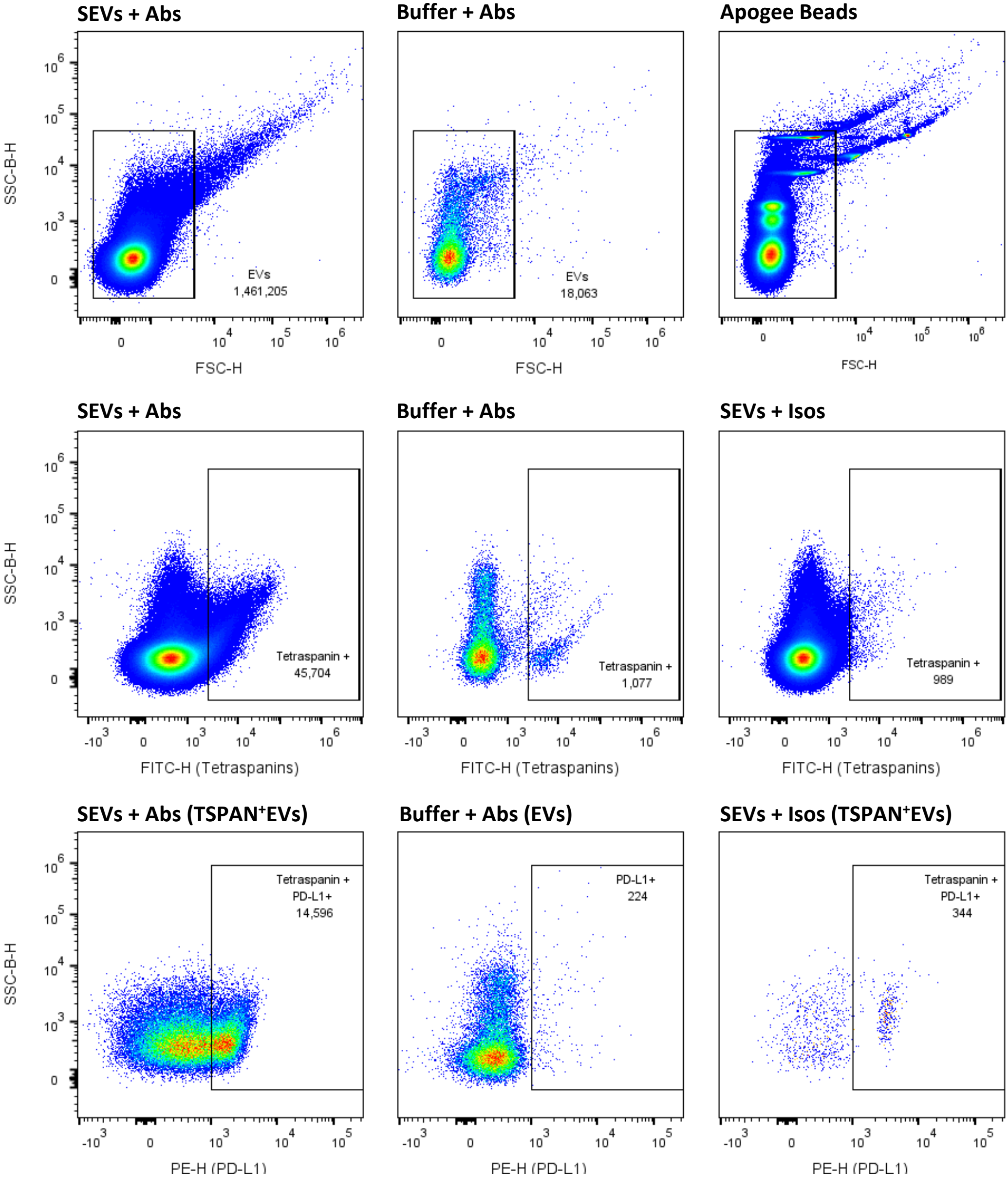

Supplementary Fig. 2. Gating strategy of the spectral-flow cytometry. First row: EVs were gated according to Apogee beads: Particles larger than 500 nm polystyrene beads were excluded. Buffer containing antibodies (Abs) was used as a control. Second row: Tetraspanin$^+$ (TSPAN$^+$) particles are gated and isotypes matching the antibodies were used. Third row: PD-L1$^+$ events within the TSPAN$^+$ population were selected, but for the buffer control only the EV gate is displayed (no TSPAN$^+$ events). The numbers represent the respective counts.

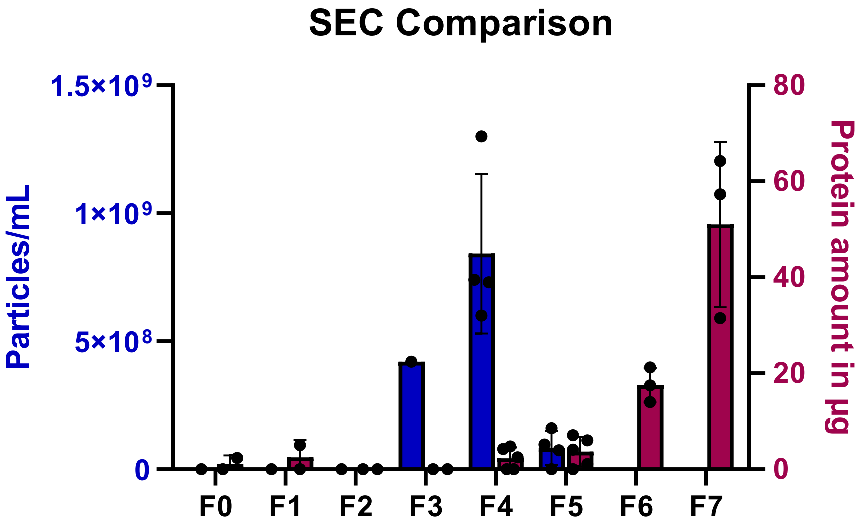

Supplementary Fig. 3. Size-exclusion chromatography fractions from saliva. Pre-cleared saliva (1 mL) was applied to a SEC column and eluted with PBS. Fractions 0-7 were collected and protein and particle concentration was measured. Most particles are eluted in fraction 4, while the highest protein yield was observed in fraction 7.

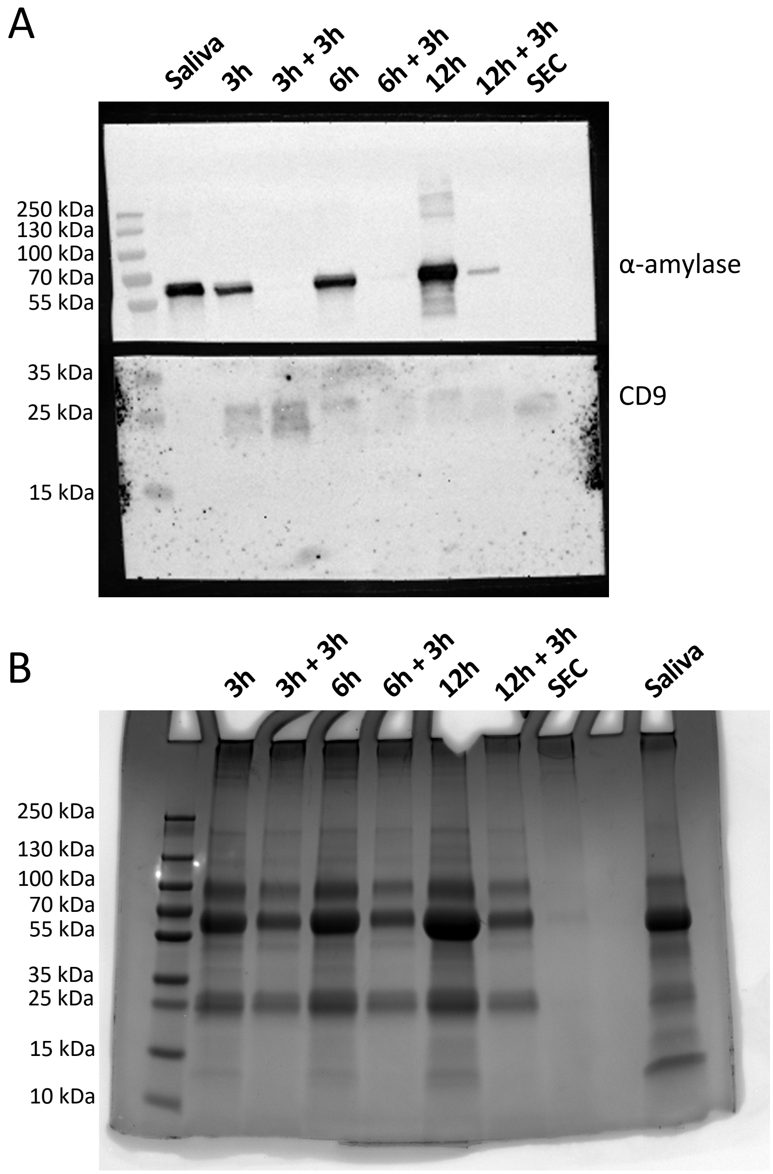

Supplementary Fig. 4. Original Coomassie stain and Western blot images. (A) Western blot of reduced α-amylase and CD9 (5 µg sample). (B) Coomassie staining of 35 µl sample per lane.

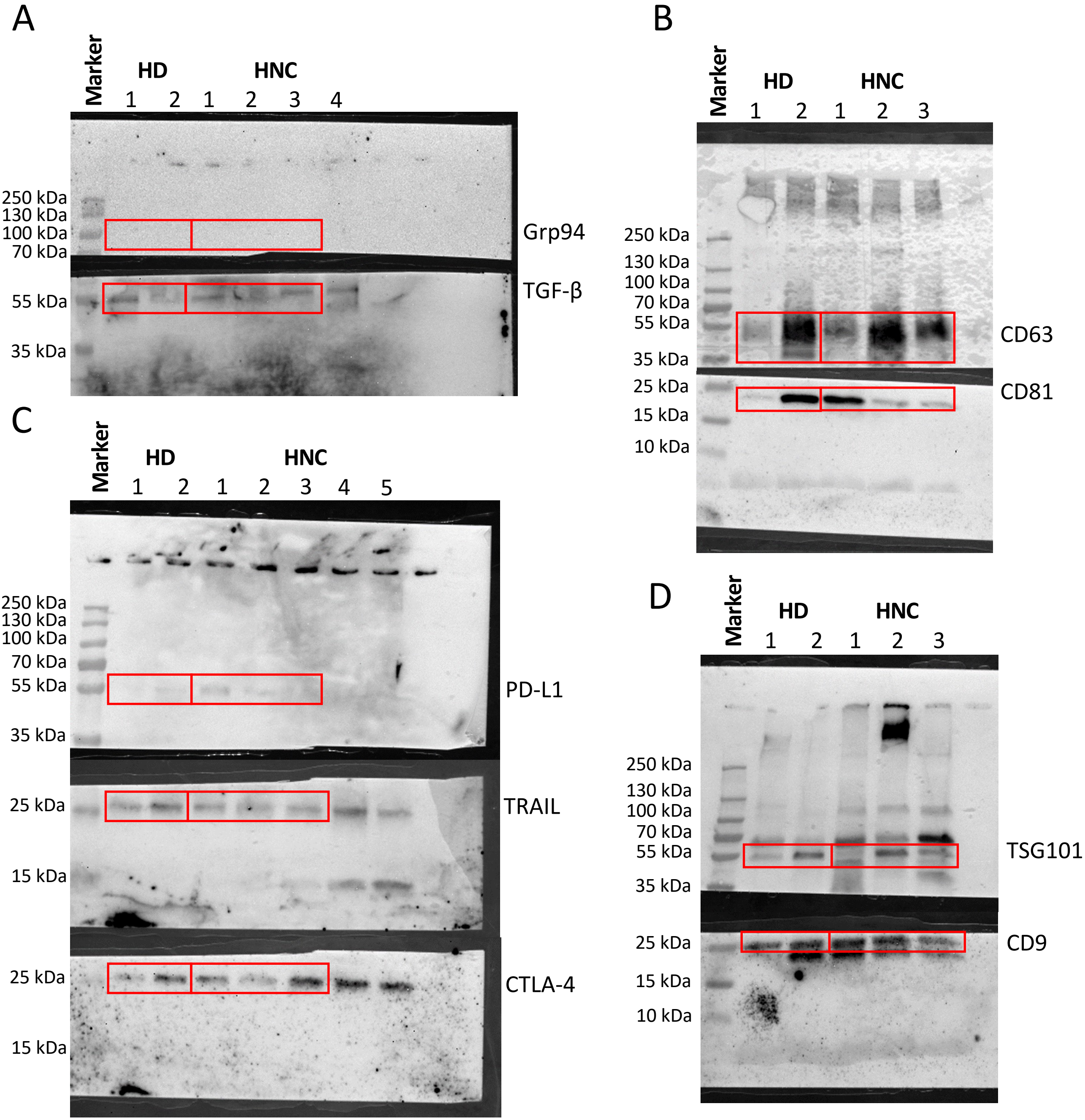

Supplementary Fig. 5. Original Western blot images. (A) Western blot of reduced Grp94 and TGF-β. (B) Western blot of non-reduced CD63 and CD81. (C) Western blot of reduced PD-L1 and TRAIL followed by CTLA-4 after stripping. (D) Western blot of reduced TSG101 and CD9.
